# Non-Wilms' renal tumors in children: experience with 139 cases treated at a single center

**DOI:** 10.1186/s12894-022-01042-3

**Published:** 2022-06-22

**Authors:** Yi Wei Fang, Hong Cheng Song, Ning Sun, Wei Ping Zhang

**Affiliations:** Department of Urology, National Children’s Medical Center, Beijing Children’s Hospital of Capital Medical University, No. 56 Nanlishi St, Xicheng District, Beijing, 100045 China

**Keywords:** Children, Non-Wilms' renal tumors, Management

## Abstract

**Background:**

Pediatric non-Wilms renal tumors (NWRTs), which comprise a small proportion of renal tumors, are a heterogeneous group of neoplasms with variable malignant potential, mortality, and response to treatment. We performed this study to determine the clinical characteristics, management and prognosis of children with Pediatric NWRTs.

**Methods:**

Medical records of all patients (n = 139) treated for NWRTs over a 12-year period (2008.01–2019.10) at a single center were reviewed retrospectively.

**Results:**

The histopathological groups of NWRTs included malignant rhabdoid tumor of the kidney (MRTK) (n: 30, 21.6%), renal cell cancer (RCC) (n: 26,18.7%), clear cell sarcoma of the kidney (CCSK) (n: 24,17.3%), congenital mesoblastic nephroma (CMN) (n: 21,15.1%), cystic nephroma (CN) (n: 16,11.5%), metanephric tumors (n: 12, 8.6%), renal angiomyoliporma (RAML) (n: 3, 2.2%), renal primitive neuroectodermal tumor (n: 2, 1.4%), renal hemangioma (n: 2, 1.4%), inflammatory myofibroblastic tumor (n: 2, 1.4%), ossifying renal tumor of infancy (ORTI) (n: 1, 0.7%). The distribution of all malignant NWRTs, including MRTK, CCSK, RCC and PNET, according to stage was as follows: stages I (n = 26), II (n = 16), III (n = 29), and IV (n = 11). The summary table shows the treatment offered to children with NWRTs. A total of 123 children were followed up for an average of 42 months. Sixteen children were lost to follow-up. Tumor-free survival was observed in 94 children. One patient who suffered from RCC is currently receiving targeted therapy and survives with the tumor. Twenty-eight children (22.8%) died.

**Conclusions:**

Pediatric NWRTs comprise 19.1% of all renal tumors in our single center. Most NWRTs can readily be distinguished using a range of immunohistochemical markers. Molecular genetic profiling has allowed much progress in the understanding of this group of tumors, making diagnosis and classification less difficult. The mainstay treatment of malignant NWRTs, including MRTK, CCSK, RCC and PNET, is comprehensive treatment. The mainstay treatment of benign NWRTs, including RAML, CN, ORTI, CMN, metanephric tumors, and renal hemangioma, is surgical resection alone and when the tumor diameter is smaller than 7 cm and the tumor locates in one pole, NSS can be performed.

**Table:** 

Treatment	Neoadjuvant chemotherapy	Nephron sparing surgery	Radical nephrectomy	Biopsy	Adjuvant chemotherapy	Postoperative radiotherapy
MRTK	2	1	27	2	26	4
RCC	1	7	17	2	1	0
CCSK	4	0	24	0	24	22
CMN	0	0	21	0	0	0
CN	0	8	8	0	0	0
Metanephric tumors	0	7	5	0	0	0
RAML	0	2	1	0	0	0
rPNET	1	0	2	0	2	2
Renal hemangioma	1	1	1	0	0	0
IMT	0	1	1	0	0	0
ORTI	0	1	0	0	0	0

*NWRTs *non-Wilms renal tumors, *MRTK *malignant rhabdoid tumor of the kidney, *RCC *renal cell cancer, *CCSK *clear cell sarcoma of the kidney, *CMN *congenital mesoblastic nephroma, *CN *cystic nephroma, *RAML *renal angiomyoliporma, *rPNET *renal primitive neuroectodermal tumor, *IMT *inflammatory myofibroblastic tumor, *ORTI *ossifying renal tumor of infancy.

## Background

Pediatric renal tumors account for approximately 6–7% of childhood solid neoplasms. Wilms tumor is the most common renal tumor in children and represents approximately 80%-90% of all pediatric renal tumors. Pediatric non-Wilms renal tumors (NWRTs), which comprise a small proportion of renal tumors, are a heterogeneous group of neoplasms with variable malignant potential, mortality, and response to treatment [1]. We retrospectively analyzed the medical records of NWRTs in our single center to assess their clinical characteristics, management and prognosis.

## Methods

### Study population

This study was carried out in the Department of Urology at a comprehensive tertiary pediatric hospital in China. Following institutional review board approval, the medical records of children (under18 years of age) with renal tumors in our hospital between January 2008 and October 2019 were retrospectively analyzed. The patient inclusion criteria required NWRTs that were first diagnosed and treated in our hospital and that the patients’ medical records were complete. Patients who were first treated in other hospitals or had no pathological diagnosis in our hospital were excluded from our study.

### Follow up and statistical analysis

All patients were regularly monitored with surveillance for the recurrence and metastasis using ultrasonography, X-ray examination and computed tomography. Overall survival (OS) was calculated from the date of diagnosis to the date of last follow-up or disease-related death. SPSS23.0 statistical software was used for data collection and analysis. OS were estimated using the Kaplan–Meier method.

## Results

### Patient demographics and clinical presentation

From January 2008 to October 2019, a total of 729 cases of pediatric renal tumors were reviewed, of which 590 (80.9%) were Wilms tumors and 139 (19.1%) were NWRTs.

The mean age of the 139 children with NWRTs was 3.5 years (range: 7 days–15 years and 2 months). The age distribution according to tumor histology is summarized in Table 1. Eighty-three patients were male, and 56 were female. The left kidney was involved in 63 cases, and the right kidney was involved in 76 cases.

**Table 1:** Age distribution according to tumor histology

Age(years)	0–1	1–3	3–5	5–7	> 7
MRTK	17	13	0	0	0
RCC	0	1	4	5	16
CCSK	3	13	5	3	0
CMN	19	2	0	0	0
CN	3	7	2	2	2
metanephric tumours	1	5	1	1	4
RAML	0	0	0	0	3
rPNET	0	0	0	0	2
renal hemangioma	0	0	2	0	0
IMT	1	0	0	0	1
ORTI	1	0	0	0	0
Total	45	41	14	11	28

*NWRTs *non-Wilms' renal tumors, *MRTK *malignant rhabdoid tumor of the kidney, *RCC *renal cell cancer, *CCSK *clear cell sarcoma of the kidney, *CMN *congenital mesoblastic nephroma, *CN *cystic nephroma, *RAML *renal angiomyoliporma, *rPNET *renal primitive neuroectodermal tumor, *IMT *inflammatory myofibroblastic tumor, *ORTI* ossifying renal tumor of infancy

Clinical presentations included 50 cases with hematuria, 37 cases detected incidentally by ultrasound examination, 34 cases with abdominal mass, 10 cases with abdominal pain, 4 cases with hematuria and abdominal pain and 4 cases with vomiting and abdominal distension. The clinical presentations according to tumor histology are shown in Table 2.

**Table 2:** Clinical presentation according to histology

Presentation	Hematuria	Abdominal pain	Hematuria and Abdominal pain	Abdominal mass	Detected by ultrasound	Vomiting and Abdominal distension
MRTK	18	2	0	8	1	1
RCC	14	2	4	3	3	0
CCSK	8	0	0	16	0	0
CMN	2	0	0	1	15	3
CN	2	2	0	6	6	0
Metanephric tumours	1	3	0	0	8	0
RAML	0	0	0	0	3	0
rPNET	1	1	0	0	0	0
Renal hemangioma	2	0	0	0	0	0
IMT	1	0	0	0	1	0
ORTI	1	0	0	0	0	0
Total	50	10	4	34	37	4

*NWRTs *non-Wilms' renal tumors, *MRTK *malignant rhabdoid tumor of the kidney, *RCC *renal cell cancer, *CCSK *clear cell sarcoma of the kidney, *CMN *congenital mesoblastic nephroma, *CN *cystic nephroma, *RAML *renal angiomyoliporma, *rPNET *renal primitive neuroectodermal tumor, *IMT *inflammatory myofibroblastic tumor, *ORTI *ossifying renal tumor of infancy

### Imaging

**Fig. 1:**
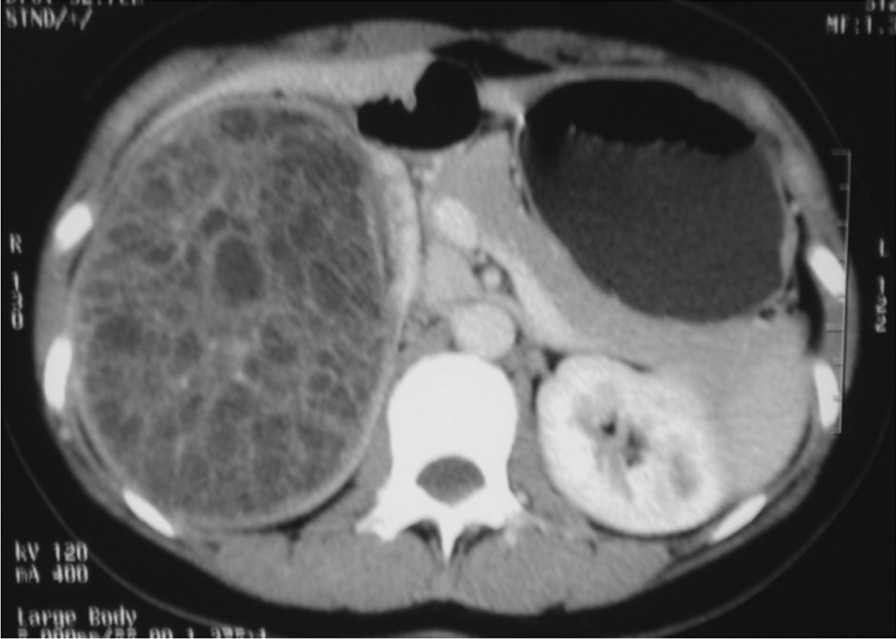
CN in right kidney: a well-defined multilocular cystic mass with an enhanced cyst septum

**Fig. 2:**
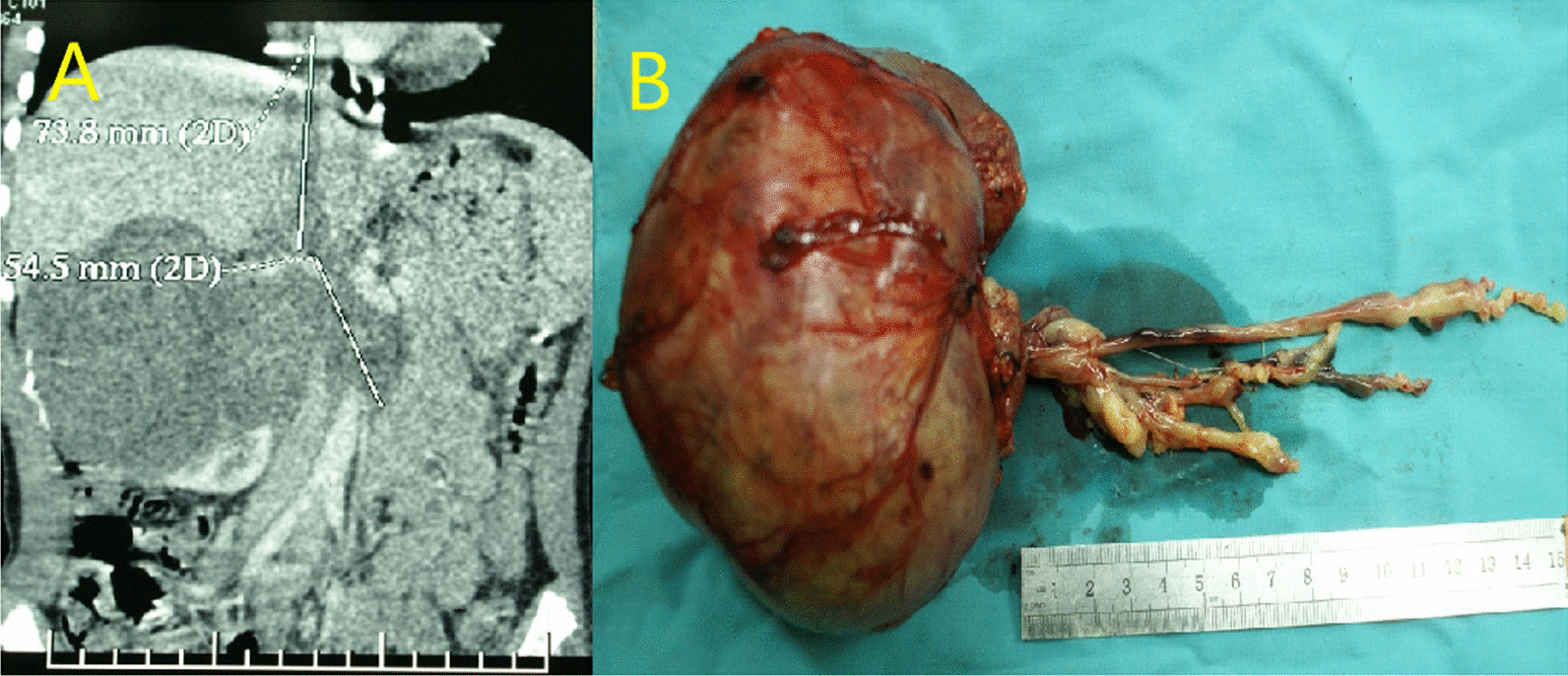
Right renal PNET combined with tumor thrombus extension through the renal vein to the right atrium **A** CT **B** Postoperative pathological specimen

Preoperative imaging, including ultrasound (US) and computed tomography (CT), was performed in all patients. The tumor sizes ranged from 2.0 × 1.5 × 1.5 to 17.7 cm × 12.8 cm × 19.1 cm, with an average of 7.5 cm × 6.1 cm × 6.9 cm. There were 100 cases (71.9%) of solid tumors and 28 cases (20.1%) of cystic tumors (Fig. 1). There were 10 cases of tumor rupture, including 5 patients with MRTK, 4 patients with RCC and 1 patient with CMN. There were a total of 9 cases of tumor thrombus, including 6 cases of renal venous tumor thrombus, 1 case of renal vein and inferior vena cava tumor thrombus, and 2 cases of tumor thrombus extension through the renal vein to the right atrium (Fig. 2A). Image-specific signs: Sixteen patients (53.3%) had typical subcapsular fluid on MRTK.

### Histopathology

The histopathological groups were malignant rhabdoid tumor of the kidney (MRTK) (n: 30, 21.6%), renal cell cancer (RCC) (n: 26,18.7%), clear cell sarcoma of the kidney (CCSK) (n: 24,17.3%), congenital mesoblastic nephroma (CMN) (n: 21,15.1%), cystic nephroma (CN) (n: 16,11.5%), metanephric tumors (n: 12, 8.6%), renal angiomyoliporma (RAML) (n: 3, 2.2%), renal primitive neuroectodermal tumor (rPNET) (n: 2, 1.4%), renal hemangioma(n: 2, 1.4%), inflammatory myofibroblastic tumor (IMT)(n: 2, 1.4%), ossifying renal tumor of infancy (ORTI) (n: 1, 0.7%).

**Fig. 3:**
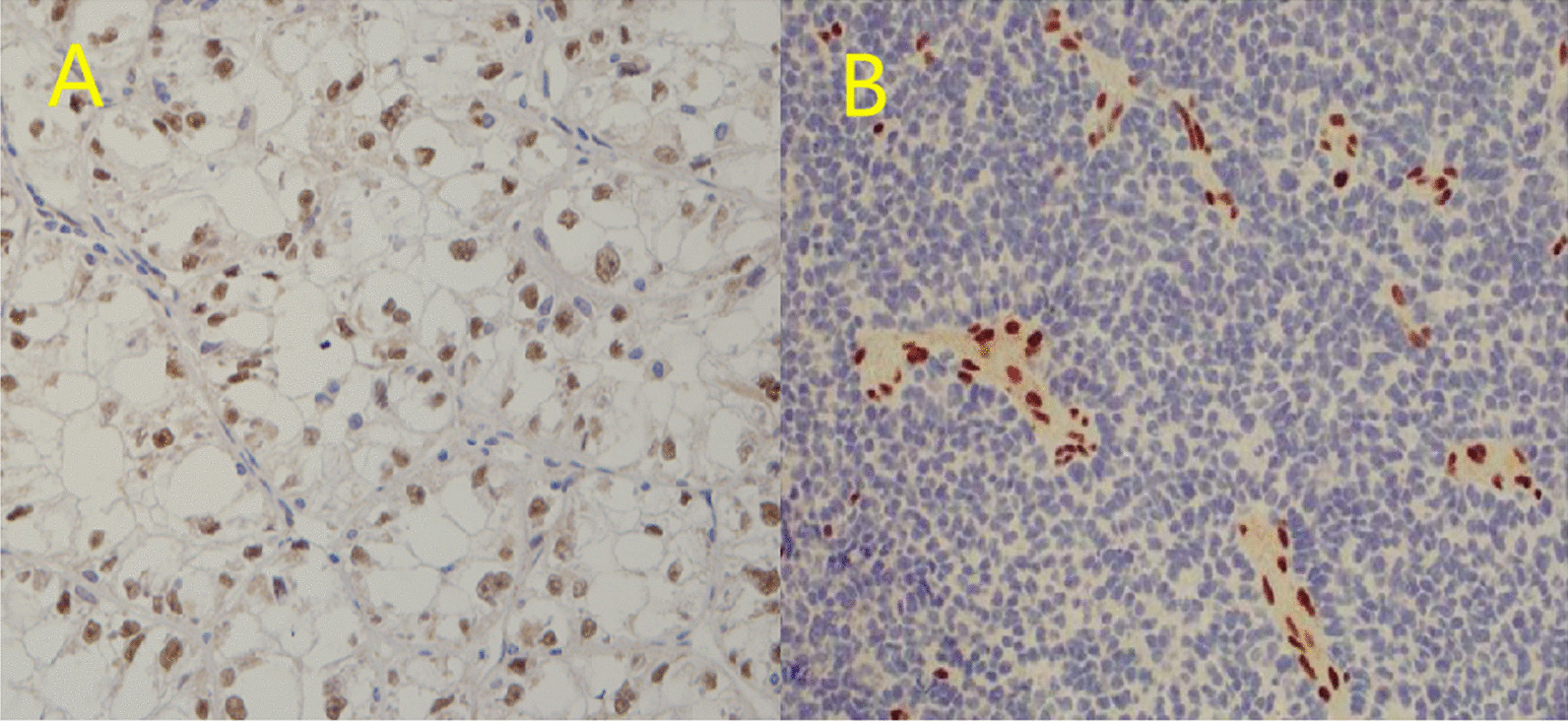
immunohistochemical assays of RCC and MRTK. **A** RCC patients underwent TFE3 immunohistochemical assays:TFE3 positive locates in the nucleus. **B** MRTK immunohistochemistry was negative for INI-1

Among the 26 patients with RCC, 20 (76.9%) had Xp11.2 translocations/TFE3 gene fusion, 3 had papillary carcinoma, 2 had clear cell carcinoma, and 1 had chromophobe cell carcinoma. All 26 RCC patients underwent TFE3 immunohistochemical assays, and 20 were positive for TFE3 (Fig. 3A). Fluorescence in situ hybridization (FISH) test was performed on 9 cases, among which 8 cases showed that the TFE3 gene locus of the chromosome had a translocation breakpoint, while 1 case of TFE3 gene locus showed no translocation breakpoint of which the TFE3 immunohistochemistry was negative. The immunohistochemistry and FISH results of the 30 MRTK cases revealed that 28 cases were INI-1 negative(Fig. 3B), 28 were vimentin positive, 23 cases were CK positive, 18 cases were EMA positive and 27 cases were Desmin negative. All the 24 CCSK cases were positive for vimentin expression and negative for neuron specific enolase (NSE), CD34, CD99, etc. Among the 21 cases of CMN, there were 7 cases of classical, cellular and mixed subtypes. There were 5 cases of metanephric stromal tumor (MST) and 7 cases of metanephric adenoma (MA) in metanephric tumors.

The distribution of all malignant NWRTs, including MRTK, CCSK, RCC and PNET, according to stage was as follows: stages I (n = 26), II (n = 16), III (n = 29), and IV (n = 11).

### Treatment and management

Neoadjuvant chemotherapy was offered to 9 patients, including CCSK (n:4), MRTK (n:2), RCC (n:1), renal hemangioma (n:1) and rPNET (n:1) which were considered for preoperative diagnosis of Wilms' tumor.

**Fig. 4:**
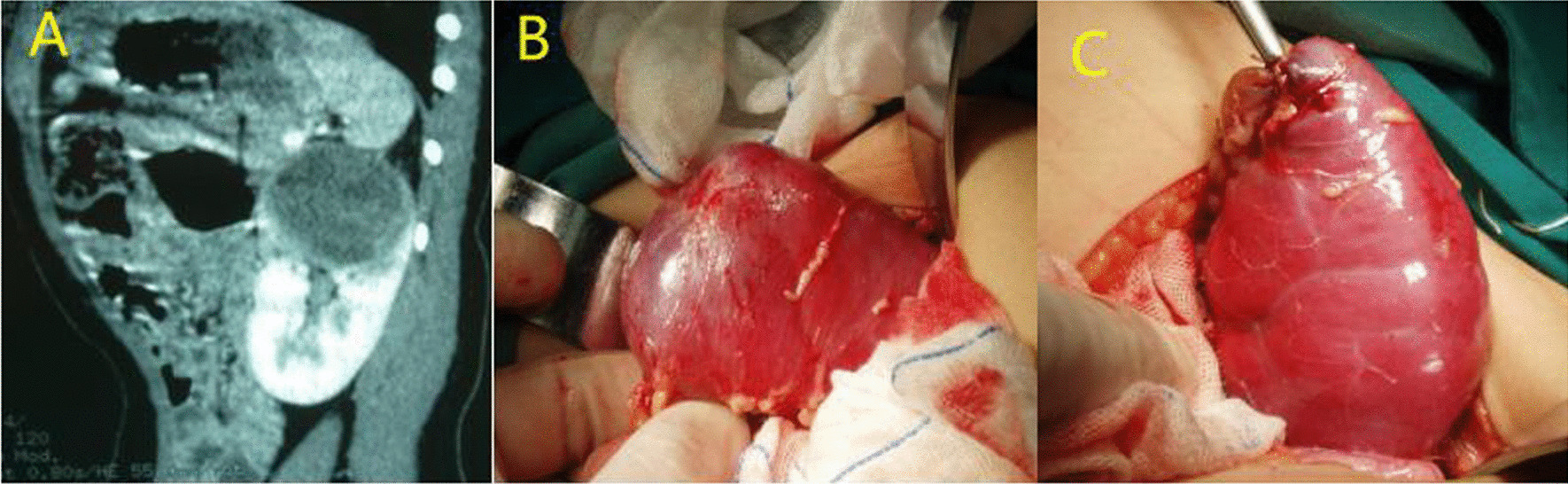
NSS performed in MRTK which the tumor located in the upper pole of left kidney. **A** The upper pole mass of the left kidney was seen in CT. **B** The tumor in the upper pole of the left kidney. **C** The left kidney after NSS

Nephron sparing surgery (NSS) was performed in 28 patients. In patients who underwent NSS, the malignant tumor diameter was less than 6 cm in all 8 cases, including 7 cases of RCC, 1 case of MRTK(Fig. 4), and the benign tumor diameter was less than 6 cm in 14 of 20 cases. A total of 107 patients were offered radical nephrectomy, including 9 patients’ tumor thrombi, which were removed en bloc with the kidney. With regards to 2 cases of tumor thrombus extension through the renal vein to the right atrium, a radical nephrectomy and thrombectomy was performed with cardiopulmonary bypass in deep hypothermic circulatory arrest (Fig. 2B). Aspiration biopsy was performed in 4 patients, including MRTK (n: 2) and RCC (n: 2).

Among the 30 MRTK patients, 4 patients gave up adjuvant chemotherapy, 26 patients were treated with chemotherapy of VDC and ICE, and 4 patients were treated with radiotherapy simultaneously. All 24 CCSK patients were treated with chemotherapy of I regimen which NWTS-5 recommended, and 22 patients were treated with radiotherapy. The 2 patients with PNET were treated with chemotherapy + radiotherapy, alternating between chemotherapy VDC and IE, and the radiotherapy dose was 36 Gy. One patient with RCC underwent targeted cancer therapy.

### Outcome

A total of 123 children (88.5%) were followed up for an average of 42 months (range 13 months to 72 months), and 16 children were lost to follow-up. Tumor-free survival was observed in 94 children. One patient who suffered from RCC is currently receiving targeted therapy and survives with the tumor. Twenty-eight children (22.8%) died.

**Fig. 5:**
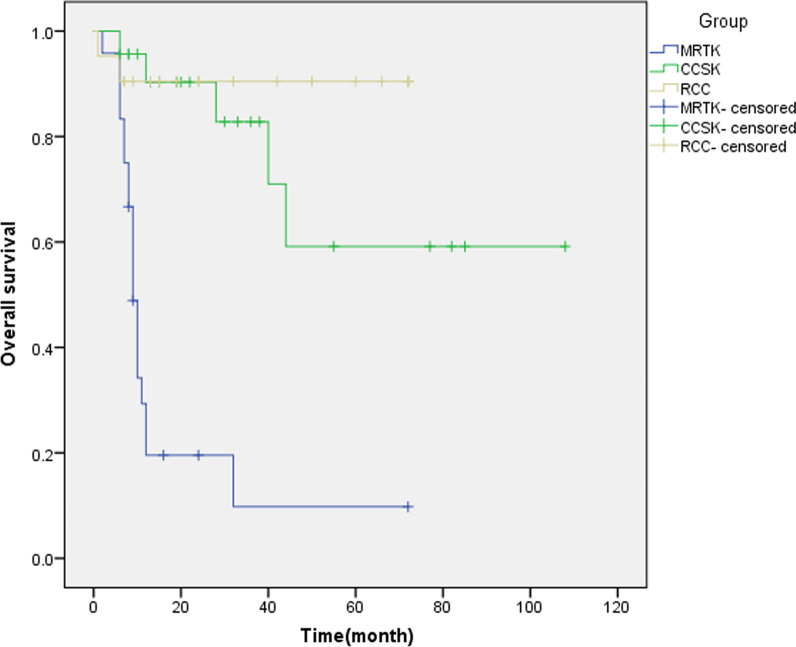
Survival of MRTK, CCSK and RCC

The follow-up of malignant NWRTs included ultrasound and chest X-ray examination every 3 months to 5 years postoperatively. Patients with MRTK had the highest mortality rate, and only 5 (5/24, 20.8%) patients survived with an average follow-up of 24 months. The patient with MRTK who underwent NSS survived more than 6 years. The overall tumor-free survival was 73.9% (17/23) for CCSK with an average follow-up of 49 months. Twenty-one of 26 patients with RCC were followed up with an average of 37 months. Nineteen patients (19/26, 73.9%) survived, including 1 patient who underwent targeted cancer therapy and 7 patients who underwent NSS. One patient with chromophobe cell carcinoma suffered from regional lymph node metastasis 6 months after undergoing NSS. Therefore, an approximately 2.5 cm × 1.8 cm × 2.4 cm mass near the left renal hilum was completely removed during repeat surgery. With a follow-up of 3 years 10 months after resection, the patient had no recurrence or metastatic disease identified. (Fig. 5). For 2 cases of rPNET, one patient presented with tumor extension through the renal vein to the right atrium and died 2.5 years after surgery due to lung metastasis; the other patient survived for 4 years until now. Therefore, the mortality rate of malignant NWRTs was 40% (28/70).

The follow-up of benign NWRTs included ultrasound examination every six months after surgery. Fifty-three of 57 patients were followed up, and no tumor recurrence was found, including 20 patients who underwent NSS.

## Discussion

Wilms tumor is the most common renal tumor in children and represents approximately 80%-90% of all pediatric renal tumors. NWRTs that occur in children include CMN, CCSK, RCC, MRTK, lymphoma, angiomyolipoma, and other rare entities [2]. Different studies have reported that the proportion of NWRTs in renal tumors varies from 13.6 to 18.7% [3, 4]. In our single center, pediatric NWRTs comprise 19.1% of all renal tumors.

### Demographics and clinical presentation of NWRTs

The patient's age at presentation, clinical features, and imaging characteristics are often sufficient to presume a diagnosis [5]. NWRTs can occur in children of all ages, but the peak age of different tumor types is distinct. MRTK is a rare but aggressive, highly malignant tumor and in general, predominantly affects young children. The International Society for Pediatric Oncology (SIOP) reported a median age of 13 months, and the National Wilms Tumor Study Group (NWTSG) reported a median age of 10.6 months [6, 7]. The median age at presentation in children with RCC is 9 years [8]. CCSK occurs with a peak incidence at 1–4 years of age [9]. CMN is the most frequent renal neoplasm of newborns and young infants, and the median age at diagnosis is 2 months [10]. CN is an uncommon benign renal lesion that occurs most commonly in children younger than 24 months of age. RAML and rPNET predominantly affect young adults in general. ORTI occurs most commonly in infancy. In our group, all the children with MRTK were less than 2 years old, approximately 62% of RCC cases were more than 7 years old, CCSK averaged 2 years and 9 months old, 90.5% of CMN cases were less than 1 year old and all RAML were more than 7 years old. Above all, children less than 6 months old are more likely to have CMN, those aged 1 to 3 years have MRTK, CCSK, or CN, and children who are 7 years of age or older are more likely to have RCC, rPNET, or RAML.

Different NWRTs have distinct presentations. Most NWRTs present with nonspecific features of an abdominal mass, abdominal pain, and hematuria, and they are detected incidentally by ultrasound [11]. In our group, hematuria was found in 50 cases (36.0%), asymptomatic ultrasound in 37 cases (26.6%), abdominal mass in 34 cases (24.5%) and abdominal pain in 10 cases (7.2%). The most commonly seen presenting symptoms in malignant NWRTs, such as MRTK, CCSK, RCC and PNET, were hematuria, which occurred in 60% of MRTK cases, 53.8% of RCC cases and 33.3% of CCSK cases. The most commonly seen presenting symptoms in benign NWRTs were abdominal masses and detected incidentally by ultrasound examination.

### Imaging of NWRTs

Preoperative imaging, which can be performed to determine the anatomic location and extent of the mass, is often sufficient to presume a diagnosis [12]. US is a good screening examination tool to determine the site of origin of a mass, to distinguish the mass between cystic or solid and to assess for possible intravascular or ureteral extension. CCSK is generally unilateral and unicentric with solid and occasionally cystic areas. Many CMN cases are diagnosed on prenatal US and can give rise to polyhydramnios, hydrops, and premature delivery. CN is a well-encapsulated multilocular tumor composed of various cysts with thin septation that compress the normal kidney. Fat content can be detected within the mass in RAML. ORTI is a well-defined, often calcified mass located in the renal pelvis and calyces. CT scans and enhanced CT scans can help to identify different tumor types. A prominent and eccentric crescent with attenuation of fluid-representing subcapsular renal hemorrhage or fluid can be identified in MRTK.In our group, 53.3% had typical subcapsular fluid on MRTK.Intravenous pyelography and radionuclide scans can be used to identify renal function. Preoperative CT scan of lung can help determine whether the tumor has lung metastasis.

### Histopathology of NWRTs

NWRTs can be diagnosed by histologic examination and distinguished using a range of immunohistochemical markers. Molecular genetic profiling has allowed much progress in the understanding of this group of tumors, making diagnosis and classification less difficult. MRTK accounts for 2% of pediatric renal tumors and consists of sheets of cells showing nuclear pleomorphism and characteristic morphologic features of open vesicular nuclei, prominent nucleoli, and scattered hyaline eosinophilic cytoplasmic inclusions. The presence of mutations in the hSNF5/INI1 gene on chromosome 22 is the hallmark of MRTK. It results in a marked reduction in nuclear expression of the gene product, which is detectable immunohistochemically [13]. In our group, The FISH results of the 30 MRTK cases revealed that 28 cases were INI-1 negative. RCC accounts for 2% to 5% of all pediatric renal tumors. RCC associated with Xp11.2 translocations/TFE3 gene fusions is the main pathological type and is characterized by chromosomal translocations involving the TFE3 gene on Xp11.2217–219 or the TFEB gene on 6p21.The most characteristic pathological manifestation is a papillary structure composed of clear cells, which is rarely seen in adult patients and is often associated with nest-shaped structures composed of tumor cells containing eosinophilic granules. Other RCC cell types include papillary renal cell carcinoma, clear cell carcinoma and chromophobe cell carcinoma [14]. CCSK accounts for 3% of renal tumors reported in NWTSG studies. CCSK has been described as soft and tan-gray in color and is well delineated macroscopically, it is composed of small round and oval cells and stellate and spindle cells that have bland nuclei. There was no characteristic immunohistochemical marker pattern, although vimentin staining was positive in CCSK. CCSK is characterized by bone and brain metastases.

CMN has low malignant potential and may exhibit several subtypes, including classical, cellular and mixed. The classical variant is composed of fibroblastic spindle cells arranged in bundles and fascicles that infiltrate the normal renal parenchyma [15]. The cellular variant is hypercellular and composed of closely packed ovoids to spindle cells with mitoses but no substantial pleomorphism. A t(12;15)(p13;q25) translocation leads to ETV6 on chromosome 12 fusing with NTRK3 on chromosome 15, which is present in the cellular variant but not in the classic subtype. CN is a well-encapsulated multilocular tumor composed of various sized cysts with thin septations that compress the normal kidney. The identifying feature of CN is that of mature well-differentiated cell types within the septa of the cyst wall. There are no blastemal or embryonal elements [16]. CN has been reported to be associated with pleuropulmonary blastoma and the DICER1 mutation. This is in contrast to adult CN, which lacks DICER1 mutations [17].

PNET is a high-grade malignant neoplasm that has a characteristic translocation t(11;22)(q24:q12) resulting in the EWS-FLI fusion gene[18]. PNET is composed of primitive-appearing undifferentiated round cells in diffuse dense cellular sheets or vaguely lobulated patterns. Metanephric tumors are classified according to the extent of epithelial versus stromal differentiation to include metanephric stromal tumors, metanephric adenofibromas and metanephric adenomas [1].RAML is the most common benign solid renal tumor, which occurs in childhood and is almost always associated with the tuberous sclerosis complex [19]. Histologically, RAML is composed of different proportions of mature fat, smooth muscle and thick-walled malformed blood vessels. ORIT has benign clinical behavior [20]. Grossly, the tumor has a nodular or irregular appearance, often partially calcified and located in the renal pelvis and calyces. ORIT is composed of osteoblast-like cells, spindle cells and an osteoid core. IMT is a rare entity that tends to exhibit aggressive behavior and local recurrence [21]. It is characterized by proliferation of typical spindle-shaped cells accompanied by inflammatory infiltration of plasma cells, eosinophils, and lymphocytes. Immunohistochemistry is positive for ALK(50–60%).

### Treatment and management of NWRTs

The treatment principle of malignant NWRTs is the need for comprehensive treatment, including surgery, chemotherapy, and, if necessary, radiotherapy [22]. SIOP reported that MRTK patients were chemosensitive to preoperative chemotherapy [6]. The extent of radical surgical excision followed by chemotherapy and radiotherapy are important determinants of long-term survival [23].In our current current present study, among the 30 MRTK patients, 26 patients were treated with chemotherapy of VDC and ICE. Since MRTK patients with hSNF5/SMARCB1/INI1 gene mutations have been identified, urgent exploration of targets for the development of novel treatment strategies is warranted in the future. As RCC is often resistant to chemotherapy and radiotherapy, no studies exist that support the use of adjuvant or neoadjuvant chemotherapy or radiotherapy. Thus, surgical resection is the mainstay of therapy. In our current present study, no RCC patient treated with chemotherapy or radiotherapy. Cook reported that NSS was suitable for children with RCC [24]. In our group, 7 cases of RCC with tumor diameters < 7 cm underwent NSS, and the postoperative follow-up showed tumor-free survival and good renal function. Therefore, we advocate nephron-sparing surgery for RCC if the mass is in polar lesions. Current therapy for CCSK includes a combination of nephrectomy, chemotherapy and radiotherapy [25]. It would possibly enhance a cure by enabling early inclusion of doxorubicin in the chemotherapy regimen. In our group of CCSK, all the patients underwent radical nephrectomy and adjuvant chemotherapy, and 22 underwent abdominal radiotherapy. Renal PNET is a rare but highly aggressive neoplasm with poor prognosis. Definite metastases occurred at the time of diagnosis in approximately 25% of patients. Effective treatment methods include a combination of surgery, chemotherapy and radiotherapy. VDC (vincristine, doxorubicin, cyclophosphamide) alternating with the use of IE (ifosphamide, VP-16) in combination is currently the recommended treatment [26].

The mainstay of treatment in most benign NWRTs is surgery and when the conditions permit NSS can be undertaken. But we suggest that NSS should be highly selective. When the tumor diameter is smaller than 7 cm and the tumor locates in one pole, NSS can be performed. CMN generally follows a benign course even with local spillage. Radical nephrectomy alone is usually sufficient; 95% of patients do not relapse, and most of the 5% who do relapse have the cellular variant of this disease [10]. However, it should be noted that none of the patients with CMN without chemotherapy had recurrence in our group. CN is adequately treated by radical nephrectomy alone, with an excellent prognosis and no chemotherapy required. With its benign nature, some have advocated NSS in polar lesions [16]. Surgery with radical excision or NSS is considered to be the treatment for metanephric tumors.In our group, 7 patients with metanephric tumors underwent NSS. For RAML, possible partial nephrectomy rather than total nephrectomy is the preferred surgical management. Angioinfarction of the tumors is also an option. ORIT has a benign clinical behavior. NSS or partial resections of the kidney may be considered. IMT is a very rare benign reactive proliferative lesion. Surgery with radical excision is still considered to be the best treatment, although steroid therapy has been reported to regress IMT [27].

The prognosis of malignant NWRTs is poor. For MRTK, age is an important prognostic indicator, as younger patients have a worse prognosis than older patients [28]. Survival for children with RCC is largely affected by the stage of disease at presentation and completeness of resection at radical nephrectomy, with an overall survival of approximately 64–87%. The NWTS reported that the 5-year relapse-free and overall survival rates for CCSK were 79–89%, respectively [29]. In our current present study, the prognosis of MRTK is the worst among MRTK, CCSK and RCC (Fig. 5). The prognosis of benign NWRTs is good. In our group of benign NWRTs, a total of 53 patients, including 20 patients who underwent NSS, were followed up, and no tumor recurrence was found.

There were several limitations to this study. First, the current present study is a retrospective, single-center study. Therefore, larger sample sizes with populations are necessary to validate the clinical characteristics of NWRTs. Second, the follow up of the current present study is an average of 42 months. A longer follow-up duration may help us to better understand the treatment, and survival of NWRTs.

## Conclusions

In conclusion, pediatric NWRTs comprise 19.1% of all renal tumors in our single center. NWRTs can occur in children of all ages, but the peak age of different tumor types is distinct. Clinical and imaging modality characteristics may support a particular diagnosis, but the final histopathologic results with immunohistochemical staining and cytogenetics should remain the gold standards for diagnosis. The treatment principle of malignant NWRTs requires comprehensive treatment, including surgery, chemotherapy, and, if necessary, radiotherapy. The mainstay of treatment in most benign NWRTs is surgery and when the tumor diameter is smaller than 7 cm and the tumor locates in one pole, NSS can be performed.

## Data Availability

The datasets generated and/or analysed during the current study are available from correspondence author Hong Cheng Song and co-correspondence author Ning Sun repository on reasonable request.

## Abbreviations

NWRTs: Non-Wilms renal tumors
MRTK: Malignant rhabdoid tumor of the kidney
RCC: Renal cell cancer
CCSK: Clear cell sarcoma of the kidney
CMN: Congenital mesoblastic nephroma
CN: Cystic nephroma
RAML: Renal angiomyoliporma
rPNET: Renal primitive neuroectodermal tumor
IMT: Inflammatory myofibroblastic tumor
ORTI: Ossifying renal tumor of infancy
